# Ex situ heart perfusion: a novel model for drug validation and translation

**DOI:** 10.3389/fcvm.2026.1790308

**Published:** 2026-06-18

**Authors:** John Onsy Louca, Magnus Althage, Nicole Asemota, Johannes Bargehr, Sai Bhagra, Dawn E. Bowles, Sarah Hosgood, Kiran Khush, Simon Messer, Marco Öchsner, Joao Pedro Nunes, Amanda Rodgers, Lu Wang, Catherine H. Wilson, Stephen Large, Sanjay Sinha

**Affiliations:** 1 Cambridge Stem Cell Institute, Jeffrey Cheah Biomedical Centre, Cambridge Biomedical Campus, The University of Cambridge, Cambridge, Cambridgeshire, United Kingdom; 2 Department of Transplantation, Royal Papworth Hospital, Cambridge, Cambridgeshire, United Kingdom; 3 Translational Science & Clinical Development Department, Research and Early Development, Cardiovascular, Renal and Metabolism (CVRM), Biopharmaceuticals R&D, AstraZeneca, Gothenburg, Sweden; 4 Department of Surgery, The University of Cambridge, Cambridge, United Kingdom; 5 Division of Surgical Sciences, Department of Surgery, Duke University Medical Center, Durham, NC, United States; 6 Department of Medicine, Division of Cardiovascular Sciences, Stanford University, Palo Alto, CA, United States; 7 Department of Cardiothoracic Surgery, Golden Jubilee Hospital, Glasgow, United Kingdom; 8 Department of Radiology, LMU University Hospital, Munich, Germany; 9 Department of Medicine, The University of Cambridge, Cambridgeshire, United Kingdom; 10 Department of Pharmacology, The University of Cambridge, Cambridgeshire, United Kingdom

**Keywords:** drug development, drug discovery, ex situ heart perfusion, first in human studies, gene therapy, novel therapeutics, organ regeneration, pharmaceutical

## Abstract

Ex situ heart perfusion (ESHP) was first developed in the 19th century by the German physician Oskar Langendorff. In recent years, ESHP has been critical to the development of donation after circulatory determination of death (DCD) programmes around the globe. ESHP has potential uses that extend far beyond transplantation. Here, we argue that ESHP, and more broadly all ex situ organ perfusion, should be utilised to perform first-in-human studies using turned down donor hearts and explanted recipient hearts from transplantation. This model would enable significantly earlier testing of novel therapeutics in human hearts, with minimal risk to patients. Widespread adoption of this model could streamline drug discovery pipelines, by enabling inefficacious therapeutics to be abandoned earlier in the drug development process. This model is particularly attractive given the high proportion of medicines that fail in stage II and stage III clinical trials due to a lack of efficacy. Development of this model will be dependent on prolonging ex situ perfusion times. Collaboration between industry, academics and clinicians will be needed to ensure successful widespread adoption of this model.

## Introduction

Ex Situ Heart Perfusion (ESHP) was first developed at the end of the 19th century by the German physician Oskar Langendorff (1). For more than 100 years, Langendorff heart perfusion has been instrumental in a wide range of biochemical, physiological and pharmacological discoveries, and is still widely used in many areas of research today (2). Normothermic ESHP has also been adopted by the heart transplant community for use clinically by enabling the use of marginal donor hearts and extending donor heart preservation times (3, 4). Furthermore, ESHP has enabled the development of donation after circulatory determination of death (DCD) programmes across the globe (5–8). DCD heart transplantation is now performed in nine countries and its application has significantly expanded the donor pool (8). ESHP uses relatively simple technology to overcome the issues associated with prolonged warm ischaemic times, and therefore enables organs to be safely utilised, with outcomes similar to conventional donation after brain/ brainstem death (DBD) transplantation (3, 9).

Circa 10,000 heart transplants take place each year worldwide (8); however, this is significantly less than the 64 million patients living with heart failure across the globe (10). Therefore transplantation is not a suitable option to treat the global epidemic of heart failure. Furthermore, heart transplantation is a blanket treatment approach for a wide range of pathologies, and development of effective therapeutics that can treat or reverse the underlying pathology are urgently required. Especially as the majority of heart failure patients are not suitable transplant candidates. Moreover, given the rising prevalence of heart failure, there is an urgent need to improve pre-clinical to clinical translation to facilitate efficient development of novel drugs (11). In this minireview we focus on the benefits and need for this new model. In addition, we discuss the necessary changes to the model as well as institutional changes to ensure that this model becomes widely adopted and well-integrated into the drug discovery pipeline.

## Difficulty of drug discovery

Drug development is a long and expensive process. The average drug takes 10–15 years to enter the market and costs between $1–2 billion (12). It is estimated that 90% of drugs that enter the clinical trials stage fail, mostly due to lack of efficacy (13). This is not surprising given the constraints of drug development. Effective therapeutics must have suitable pharmacokinetics and pharmacodynamics, a suitable safety profile, and maintain efficacy on top of these constraints. However, therapeutics are predominantly tested in cell lines and animal models. Cell lines are simplistic and lack the complex interactions found in human organs. They enable the effect of a drug on an individual cell type to be specified, but lack the complex physiology and interactions seen in human organs. Even more complex *in vitro* models such as organoids still lack the complexity of fully developed human organs and do not replicate disease states for complex multi-system diseases such as heart failure. Thus, they have their use early on in drug development for large-scale screening of molecules but have limited use beyond this. Whilst animal studies have their use, especially in allowing testing on complex multi-system organisms, there is still a need to test novel therapeutics in human translatable models at significantly earlier stages of drug development. In a 2017 opinion piece on phenotypic drug discovery, Moffat and colleagues discuss the importance of a ‘chain of translatability’ and point to a key limitation of animal models—that even in circumstances with similar molecular drivers of disease, the resultant pathogenic mechanism may be fundamentally different (14, 15). Moreover, animal models often lack the full complexity of comorbidities seen in many patients (such as obesity, diabetes, and hypertension) such that there may not be suitable animal models for a given disease. Thus, whilst animal studies are useful, they have multiple shortcomings which may partly account for the high failure rate seen in clinical trials due to lack of efficacy. Importantly, drug development today aims to Replace, Reduce and Refine the use of animals in research, according to the 3Rs principle (16).

Therapeutic development failure is particularly important in the development of cardiovascular drugs, which have the second highest failure rate, second only to oncology drugs (17). Therefore, there is a clear need for testing of novel therapeutics in clinically relevant human models. Clinical trials are expensive, with the costs of phase I, II & III trials estimated to be $3.4 million, $8.6 million and $21.4 million respectively (18). ESHP provides a safe human model, with minimal risk to patients, and with various pathologies across a range of severities, that could be tested significantly earlier on in the drug discovery pipeline to assess efficacy and safety. Crucially, the potential of human ESHP remains largely untapped in drug development, with little work carried out to date.

## Previous uses of ex situ heart perfusion in therapeutic testing

**Figure 1:**
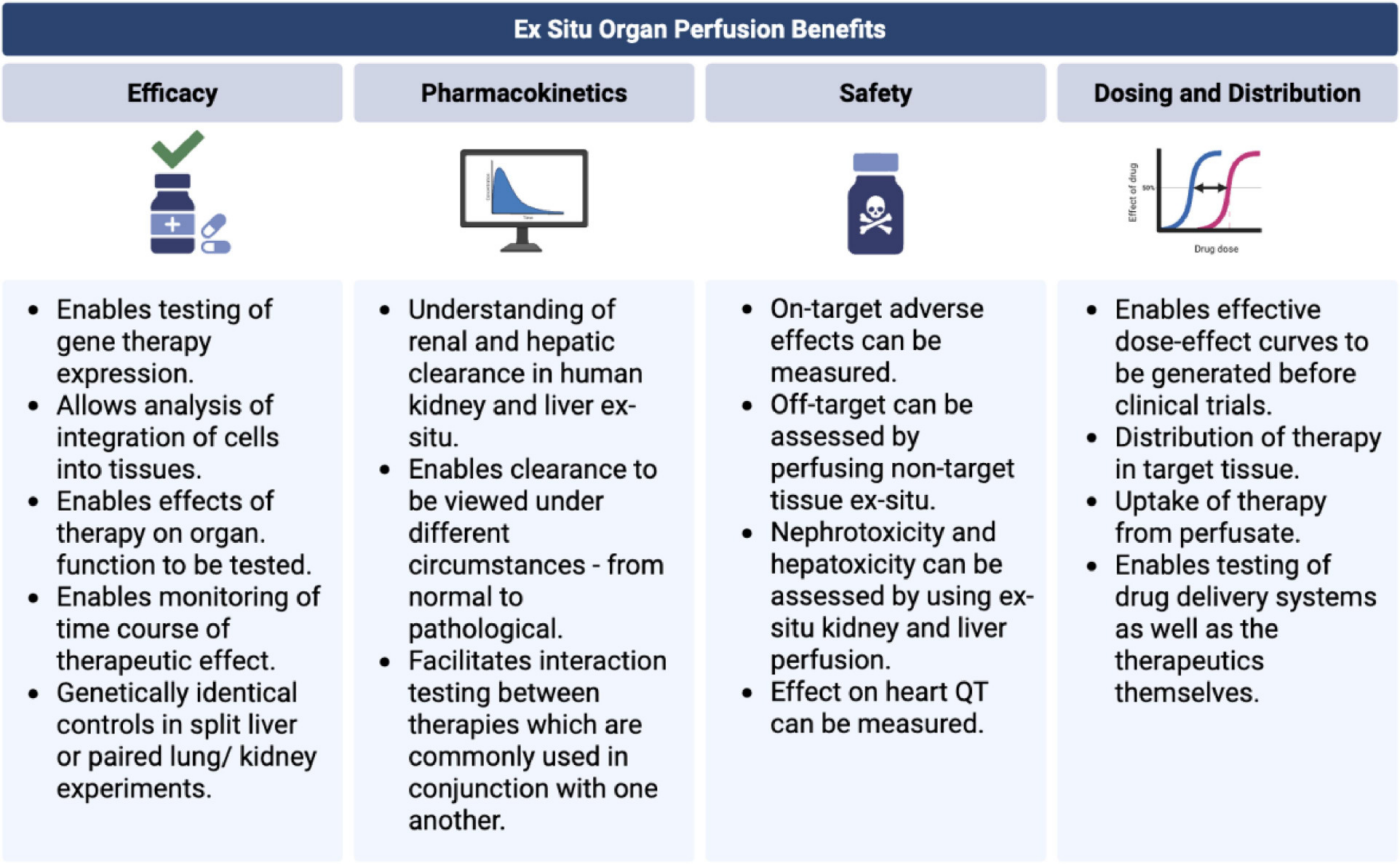
The benefits of Ex situ organ perfusion for use in turned down donor hearts and explanted recipient hearts. Summarises the main benefits of ex situ organ perfusion as a model in drug discovery using both turned down donor organs and explanted recipient organs. These benefits are universal to all organs, with the added benefit of pharmacokinetics being able to be assessed in livers and kidneys.

Whilst ESHP has been performed for more than a century, it has not yet been extensively used in human hearts for validating novel therapeutics, with only proof-of-concept studies having been performed up until this point. We previously collaborated with colleagues at AstraZeneca & Moderna to test a novel vascular endothelial growth factor-A (VEGF-A) mRNA. This molecule had previously shown promise in a phase II clinical trial (EPICCURE study) with potential improvements in quality of life and heart function in patients undergoing coronary artery bypass grafting (19). However, assessment of VEGF-A protein production in human myocardium was not possible, nor was the distribution or optimal dose established. We therefore utilised ESHP on explanted recipient hearts to test the expression of A (VEGF-A) protein following injection of a novel VEGF-A mRNA (20). Our study demonstrated expression of VEGF-A protein derived from this novel mRNA in the different layers of the heart, revealing that there were significantly elevated VEGF-A protein levels in the subendocardium and mid-myocardium. We also demonstrated higher levels of VEGF-A protein with low-dose compared to high-dose mRNA. Whilst the study was limited by the small numbers of available hearts and downstream analysis performed, certain types of cardiomyopathies (dilated and ischemic) appeared to provide environments that enabled more efficient translation of the therapeutic mRNA compared to other types of cardiomyopathies (hypertrophic). This proof-of-concept study provided insights into dosing, drug distribution, efficacy and timing of expression of this mRNA. Importantly, the study demonstrated the potential utility and benefit of this model as a translational system (Figure 1), by being part of the development of novel medicines for heart failure to be adopted in clinical practice. However, at the time of writing this review this has been the only study which has utilised ESHP on explanted recipient hearts to test a novel therapeutic.

ESHP is also being utilized as a platform to deliver advanced therapeutics (including gene therapies) to donor hearts prior to transplantation (21). To date, these studies have utilized the firefly luciferase marker gene (luciferase) in Adenoviral (Ad) and adeno-associated viral (AAV) vectors to evaluate feasibility, duration and biodistribution of gene delivery and expression. They have also been used to test safety in heterotopic porcine heart transplant models (21–23). ESHP has the advantage of creating a metabolically favourable environment for vector transduction, whilst isolating the heart from other organs and therefore minimising the risk of systemic side effects from gene delivery to unintended organs. Previous work has demonstrated robust and widespread gene expression in the donor hearts up to 4 months post treatment during ESHP, with no evidence of vector derived genetic material or gene expression in recipient native organs such as liver (22, 23). Both studies utilizing AAV based vectors have demonstrated sustained gene expression in the donor heart, with Dewan et al. recently reporting robust gene expression up to 120 days with no evidence of functional or structural impairment of the donor organ (23). Delivery of therapeutic genes to donor hearts via ESHP prior to transplantation may eventually be used to improve cardiac allo-transplant and xeno-transplant outcomes as well as to enable repair of genetically-triggered cardiomyopathies through auto-transplant (24). This is a radical re-imagining of the use of ESHP, which has the exciting potential to extend beyond simple graft preservation in heart transplantation.

## Ex situ organ perfusion for other organs

Kidneys, livers and lungs have all been used in ex situ organ perfusion studies to assess efficacy of novel therapeutics (Table 1). Using turned down human livers that were perfused for 100 h ex-situ, a group in Cambridge has successfully demonstrated the efficacy of cholangiocyte organoids in repairing bile duct epithelium (25). This land-mark study effectively demonstrated that cholangiocyte organoids could be used to treat various biliary tree pathologies. Importantly, it demonstrated the benefits of perfusing organs for extended durations and the ability to manipulate both physical and biochemical parameters in such experiments. Turned down donor livers perfused ex situ have also been used to determine rAAV gene therapy expression and engraftment of multipotent adult progenitor cells (26, 27). Other groups have demonstrated the potential use of multipotent adult progenitor cells used to treat turned down donor kidneys on normothermic perfusion machines (28). These kidneys demonstrated increased urine output and improved microvascular perfusion. Another study in kidneys showed the potential use of anti-sense oligonucleotides in targeting deleterious micro RNAs, however this was a small proof-of-concept study (29). Work in kidneys has also demonstrated that ex situ organ perfusion may be used to test therapeutic delivery methods as well as therapeutics themselves (30). Moreover, others have demonstrated the potential utility of ex situ lung perfusion from turned down donor lungs to test various cell and gene therapies to optimise graft function (31–33). One advantage realised in some of these studies is the ability to use a genetically identical organ as a control – whether it be with pairs of donor kidneys, or split liver or split lung experiments (26, 28, 32). It may also be possible to use the heart as its own control as we demonstrated previously. There were control injection sites which did not have the mRNA injected, and ‘test’ sites where the mRNA was injected. This is a significant advantage that has not yet been widely realised with this model. A true, genetically identical control is virtually non-existent in clinical studies with the exception of twin-studies. Together, these studies give insights into the potential utility of ex situ organ perfusion to evaluate both gene and cell therapies. Moreover, they demonstrate that this technology has already been successfully leveraged in various models by different groups. Heart transplant teams would do well to replicate this success.

**Table 1:** A summary of the different uses of ex situ organ perfusion in drug discovery.

Organ	Duration of perfusion	Pathology	Result	Reference
Heart	8 h	Explanted transplant recipient hearts with ischaemic or dilated cardiomyopathy.	Validation of protein expression in target tissue.	Louca et al. (20)
Liver	100 h	Turned down donor livers with ischaemic duct injury.	Gallbladder organoids were able to integrate into and repair bile ischaemic bile duct injury.	Sampaziotis et al. (25)
Liver	12–16 h	Turned down donor livers – one due to sepsis and the second due to old age. These livers were split, so one half acted as the control and the other half as the study arm.	Insights into the kinetics of AAV transduction as a vector for gene therapy	Cabanes-Creus et al. (26)
Liver	6 h	Turned down donor livers.	MAPCs exert anti-inflammatory effects on livers perfused ex situ.	Laing et al. (27)
Livers	25 h	Turned down livers due to steatosis.	Hepatic clearance of 2,4 – dinitrophenol delivery characterised	Tingle et al. (35)
Kidney	7 h	5 pairs of turned down donor kidneys due to donor non-renal malignancy. This enabled one kidney to be the control and the other to be the study kidney.	MAPCs increase urinary output, improve microcirculation flow and exert anti-inflammatory effects.	Thompson et al. (28)
Kidney	6 h	Turned down donor kidneys.	Profiling of miRNA expression during ESMP and proof of concept that ASOs can be used to target possibly deleterious miRNAs.	Thompson et al. (29)
Kidney	4 h	8 pairs of turned down donor kidneys. This enabled one kidney to be the control and the other to be the study kidney.	Anti-CD31 tagged nanoparticles accumulate in the endothelium of kidneys, suggesting a potential use of a drug delivery system.	Tietjen et al. (30)
Lung	12 h	Turned down donor lungs.	IL-10 gene therapy delivered ex situ had beneficial effects on lung function and could potentially enable transplantation of marginal donor lungs.	Cypel et al. (31)
Lung	12 h	Turned down donor lungs. The lungs were split and one acted as control and the other as the study arm.	Gene modified MSC application resulted in increased IL-10 levels, but this was less effective in more damaged lungs, due to an acidotic tissue microenvironment.	Nykänen et al. (32)
Lungs	6 h	Turned down donor lungs.	Use of MSC microvesicles improved alveolar fluid clearance and decreased lactate levels in the perfusate.	Gennai et al. (33)

TABLE 1 summarises the different uses of ex situ organ perfusion. Most of these studies have been proof of concept studies, but they demonstrate the potential utility of this model in drug discovery in a range of organ systems.

AAV, adeno-associated viruses; ASOs, anti-sense oligonucleotides; MAPCs, multipotent adult progenitor cells, MSC, mesenchymal stromal cells.

## Heterogeneity of ex situ organs

One of the significant advantages of Ex Situ Organ Perfusion is the use of both turned-down donor and explanted recipient organs. These represent distinct groups of organs, each with particular advantages. For instance, turned-down donor hearts provide a model with relevant comorbidities seen in much of the general population, in a predominantly middle-aged and older adult population. These non-failing hearts represent a more ‘normal’ heart – i.e., more similar to that of an individual in the population without cardiomyopathy. These hearts would be useful for testing biodistribution of novel therapies or vectors without the confounding variables of diseased myocardium. They could also be used for testing pharmacodynamics, pharmacokinetics and safety. Moreover, turned-down donor hearts could be used to test therapies for acute injuries – e.g., in a myocardial infarction model, with therapeutics aimed at reducing ischaemic-reperfusion injury. Recipient organs, on the other hand would be useful for studying specific disease phenotypes and end-stage organ failure. This is particularly attractive for regenerative therapies aiming to reverse/ repair underlying pathological processes. In addition, recipient hearts offer an attractive model for specific genetic mutations. Genetic testing is becoming more widely adopted in patients with cardiomyopathy and several gene variants are well-established to cause cardiomyopathy (34). These organs offer an attractive and obvious target for gene therapies to reverse the underlying pathophysiology. Theoretically it is possible that both groups of organs could be used in a single study, with turned-down donor organs representing ‘healthy controls’ and recipient organs acting as the desired treatment group. Such a model is uniquely powerful because it incorporates the broad heterogeneity of the human population. By reflecting diverse genetics and environmental exposures, ex situ organ perfusion accounts for the real-world variation in drug efficacy, a critical factor that cell and organoid models cannot yet accurately replicate.

## Ex situ organ perfusion use in safety testing

Expanded use of ex situ perfusion to livers and kidneys would also enable safety testing from a nephrotoxic and hepatotoxic standpoint as well as enable quantification of both renal and hepatic clearance (35). In hearts, QT and QTc testing could be performed ex situ. Although more work will be needed as the QT interval is not recorded on hearts perfused ex situ, nor is the effect of ex situ heart perfusion on QT duration well documented. Nonetheless, this model may offer a valuable opportunity to study QT intervals, which is of great importance in drug development (36). This model may have the potential of being more translatable than *in vitro* assays and animal models and may therefor improve development of safer and more efficacious drugs to patients.

## Current limitations of ESHP

**Figure 2:**
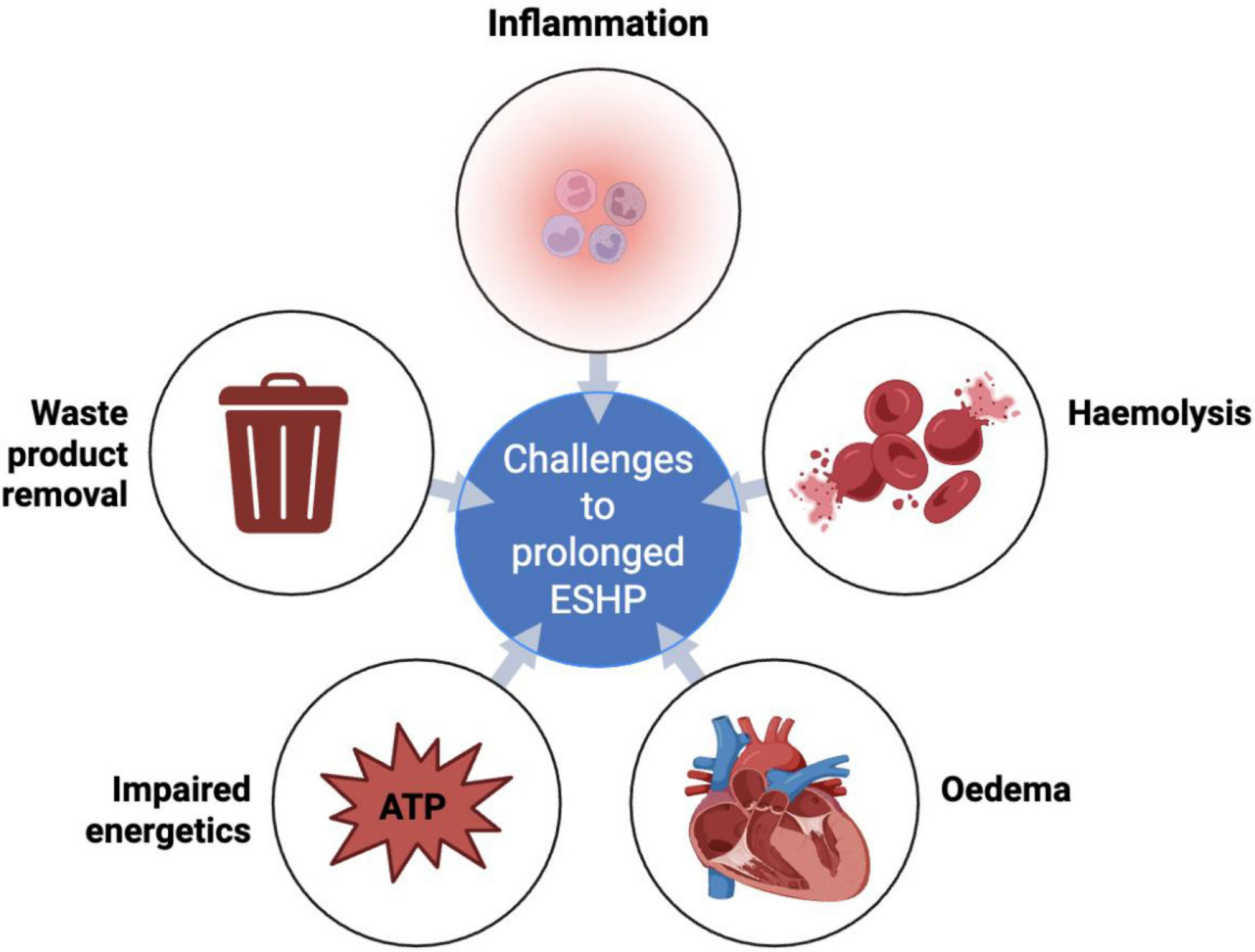
Challenges to overcome and potential solutions when using ESHP for therapeutic drug development. Summarises the 5 main challenges that must be overcome to successfully increase the duration of ESHP. There has been research by different groups across using both clinical and preclinical models into these challenges, however as of yet long-term ESHP has not been achieved. Ongoing work is needed to provide a definitive method of prolonging the duration of ESHP.

We believe ESHP offers a translational opportunity in drug development for testing efficacy, dosing, distribution, pharmacokinetics and pharmacodynamics in a variety of pathogenic (explanted recipient) and more ‘normal’ (turned down donor) hearts. However, the utility of ESHP as a model is currently limited by two factors. The first is the short perfusion times. Hearts perfused ex-situ demonstrate a continuous clinical deterioration in both systolic and diastolic function, and perfusion duration is typically limited to 6-hours (4, 37). This deterioration has been described clinically and experimentally (38). Attempts have been made to prolong ESHP. The longest clinical run has been documented in Kazakhstan where a donor heart was perfused for 17 h prior to transplant with the recipient still alive 3-years post-transplant (39). However, the duration that a heart can be supported ex situ falls short of the perfusion periods that have been achieved with abdominal organs. Livers are regularly perfused for >100 h, with perfusion having been extended to 7 days in human livers (25, 40). Therefore, to increase the utility of the heart model there is a need to prolong perfusion times. This will allow not only for the immediate effects of a therapy to be tested (such as protein production following mRNA administration) but also the mid-term effects (i.e., the effect of this protein on cellular and organ function). However, there are several challenges that must be overcome before ex situ heart perfusion can be successfully extended (Figure 2).

- Integrating renal replacement therapy into ESHP circuits. It has previously been demonstrated that pig hearts perfused ex situ retain both better systolic and diastolic function as well as improved coronary vasomotor function at 8 h when dialysis was used compared to controls (41). It has yet to be widely used clinically, mainly due to the short perfusion times used. Removing waste products and potentially excess water by using dialysis is a viable method of improving heart function and easily implemented.
- Reducing haemolysis of the ex situ perfusion circuit. Haemolysis is a well-documented complication seen in extra-corporeal circuits (42). Haemolysis presents a challenge due to the toxic nature of free haemoglobin on endothelium, which acts to decrease NO levels, disrupt the endothelial barrier and active endothelium (43). Therefore minimising haemolysis, by optimising circuit configuration as well as by scavenging free haemoglobin may reduce endothelial injury and improve heart function ex situ (44).
- Reducing the inflammatory response seen in response to ex situ organ perfusion. Blood that is exposed to air or comes into contact with plastic tubing displays an inflammatory response. This has been well documented in extra-corporeal membrane oxygenation (ECMO) circuits (45). A strong inflammatory response has previously been characterised in pig hearts (46). There are several possible methods of overcoming this inflammation. Removal of white cells as is done clinically on the Organ Care System by use of a white cell filter may reduce inflammation – however there has been no published report demonstrating the efficacy of this filter in reducing inflammation or improving heart function and clinical outcomes. Cytokine adsorbers are an alternative and early data seems encouraging with the ability to remove a wide range of inflammatory mediators (47).
  An alternative method of reducing inflammation would be to simply remove the white blood cells mediating the inflammation. It is well established that acellular Langendorff perfusion is feasible in both small and large animal models (48–51). It is still not clear whether a blood-based or acellular perfusate is optimal for long-term perfusion. Work from the Freed lab demonstrated that in an ex situ preparation of pig hearts that a whole blood-based perfusate was associated with several advantages. This included superior preservation of function, reduced oedema and reduced methaemoglobinemia compared to an artificial haemoglobin carrier (49). However, they demonstrated that whole blood resulted in significantly lower rates of inflammatory mediator release compared to erythrocytes alone, suggesting a role for plasma proteins in inflammation. However, more recent work has demonstrated that acellular perfusates with adenosine resulted in lower rates of Troponin I, Myosin Light Chain 3 and interleukin 6 release in a rat model of ex situ perfusion (50). It is not clear which of these methods is optimal and more work, including a better understanding of the molecular mechanisms mediating inflammation ex situ are needed.
- Optimising the pressure and flow of perfusion. Heart perfusion is complicated beyond normal organ perfusion as the heart may either be perfused in simple Langendorff (also known as non-working mode, with an empty left ventricle) or working mode (with a left ventricle full of blood, pumping blood out via the aorta). Clinically hearts are only perfused in non-working mode, i.e., in a simple Langendorff perfusion set-up. It is still unclear which method is superior, and it is worth noting that perfusion in working mode is complex and requires additional expertise and modification of circuits. However, working mode enables superior assessment of heart function, for example using pressure-volume loops – the gold standard for assessing heart function in a load independent manner (51). Beyond this, it is still unclear what the optimal pressure and flow is required for ex situ heart perfusion. Work by colleagues in Canada has demonstrated that perfusing with flow control rather than a pressure control system improved heart function and was associated with reduced markers of oxidative stress (52).
- Optimising the perfusate. There is little published work on how best to optimise the perfusate of hearts perfused ex situ. A key component of the perfusate is how best to provide energy for the heart. Recent work has demonstrated that perfusing hearts with a low-glucose, ketone-rich perfusate reduces oedema and improves heart function. Further work is needed to understand what other metabolites are needed and whether further adjuncts such as antioxidants or other drugs further improve ex situ heart perfusion (53). More recent work has demonstrated the potential utility of further optimising perfusion ex situ by addition of T3 or by the use of ischaemic pre-conditioning. Therefore, optimising the perfusate has the potential to extend far beyond metabolism and includes well-established and potentially novel therapeutics to supplement the perfusate (54, 55).

Prolonging ESHP is feasible but will require a systematic approach to address the above challenges.

In the UK and the US, the overwhelming majority (99.7% and 88.7% respectively) of turned down donor hearts are not used in research (56, 57). The reasons for this are unclear, but mostly likely due to the physiological factors associated with the heart, logistical issues and a broader lack of awareness of the potential use of these in research. Hearts are sensitive to ischaemia – more so than kidneys and livers (58). Within 30-minutes of warm ischaemia, hearts need to be explanted and placed on ice or ex situ perfusion machines. Moreover, in the UK the majority of organ donors do not donate thoracic organs for clinical use – due to strict clinical requirements. Therefore, cardiothoracic teams do not attend most organ retrievals, thus limiting the number of hearts that are available to be used in research. In the US, there is significant variation in the rates of organ retrieval for research depending on the organ procurement organisation (57).

## Next steps for wider implementation of ex situ organ perfusion as a tool for novel drug development

Ex situ organ perfusion has great potential to boost the productivity and efficiency of drug discovery pipelines, with novel therapeutics tested on both pathological and ‘normal’ human organs significantly earlier on in their discovery.

There is a need for close collaboration between academics, industry and clinicians to ensure that organs are effectively used for drug discovery and organ regeneration research. Local policy makers should engage and provide frameworks for this research and guidance on how this model should be used to complement the findings in clinical trials.

Logistical issues such as the lack of cardiothoracic retrieval teams can be overcome. Abdominal teams can be trained to procure hearts with relative ease and indeed in some centres abdominal teams are trained to procure DCD hearts. Alternatively, a dedicated research retrieval team in each region working with local centres would be a viable and inexpensive option to retrieve these hearts for research. Moreover, this would have the benefit of increased training in cardiothoracic retrieval for more junior members of the team in a relatively ‘low stakes’ retrieval. Moreover, closer collaboration between industry, clinicians and academics will build both infrastructure and ensure sufficient funds are available for this model.

Engagement with relevant stakeholders including family members of organ donors, patients with organ failure and transplant recipients is essential to ensure that appropriate conversations regarding the use of these organs in research are carried out. Preliminary work with these groups has shown strong support for use of this model, especially given its potential widespread benefit to patients (59). Moreover, communication needs to be two-way, and clinicians and academics should clearly convey the benefits of this research and as this becomes more widely adopted will be able to point to successful studies using this model (60).

Perfusion machines used clinically incur a significant expense and are often run by third party companies meaning that expertise on running these machines may not be readily available in some transplant centres. Therefore, there is a need for an effective organ perfusion machine that is cost-effective and can facilitate such studies with in-house expertise. In our experience, such studies require input from clinicians including transplant surgeons and donor organ physiologists as well as academics and scientists from the pharmaceutical industry who provide expertise on the therapeutics.

Despite the challenges, ESHP provides great value for translational work, even with short perfusion times. It provides information on immediate effect, dosing and distribution that could never be achieved in patients and can provide information on renal and hepatic clearance as well as preliminary safety information.

**Figure 3:**
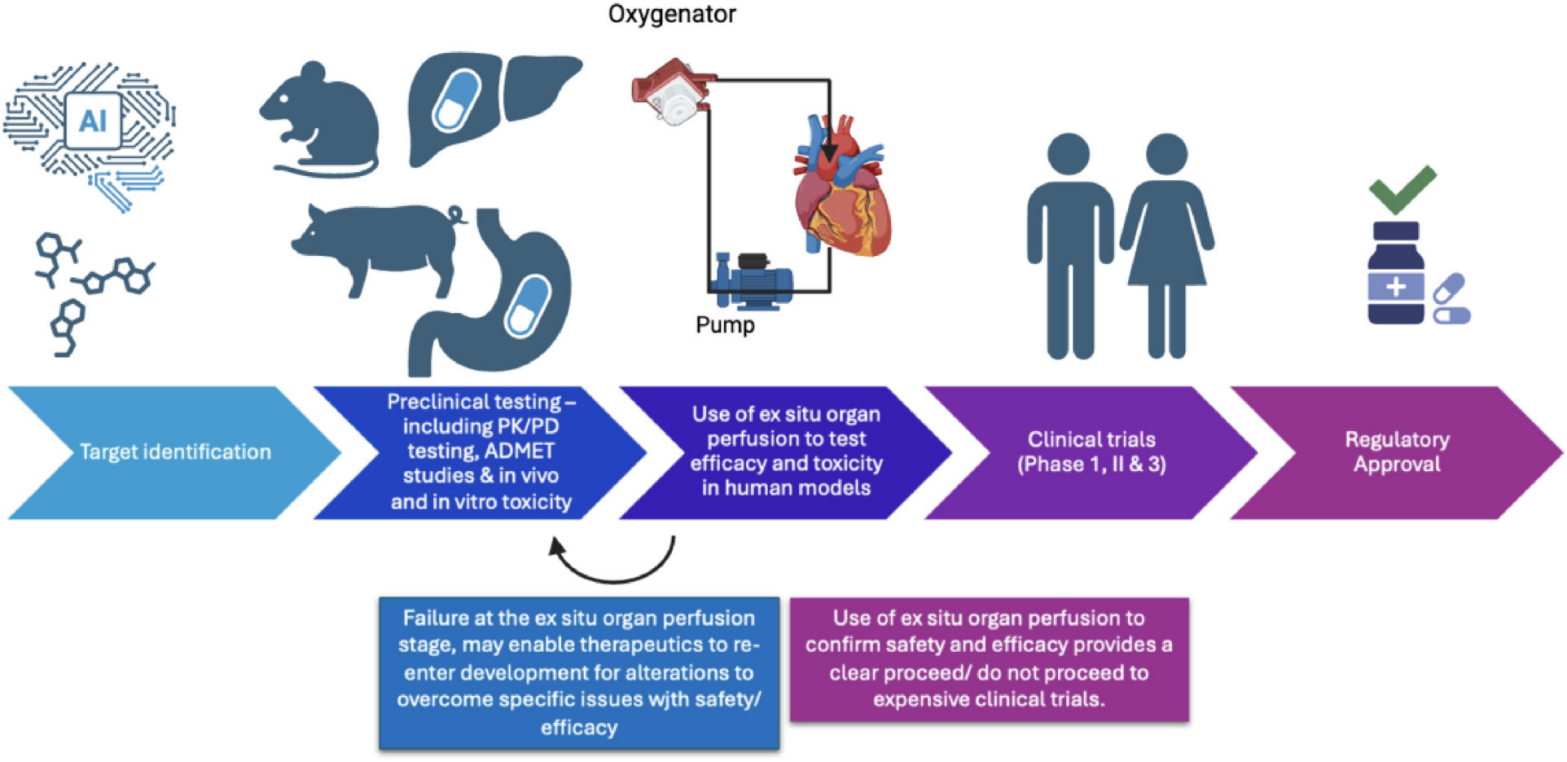
The drug discovery pipeline, incorporating the use of Ex situ heart perfusion for efficacy and toxicity testing before the clinical trial stage. A proposed drug discovery pipeline that incorporates efficacy testing using turned down donor and explanted recipient organs both for toxicity and efficacy of novel therapies. PK, pharmacokinetics; PD, pharmacodynamics; ADMET, absorption, distribution, metabolism, excretion and toxicity. The organ perfusion stage would act as a clear go/ no-go signal for clinical trials. Moreover, in the case of unsatisfactory results from the organ perfusion testing, alterations can still be made to the therapeutic with further testing carried out subsequently.

Figure 3 demonstrates where we propose this model could be used in the drug development pipeline. ESHP would ultimately be the last step before clinical trials, given its low-throughput nature and therefore the comparatively small number of compounds that could be screened when compared to *in-vitro* and animal studies. Use of ESHP to prevent one therapeutic from progressing to phase III clinical trials would save in the region of $33 million.

ESHP promises to be a mainstay of drug development, especially as ex situ organ perfusion continues to improve. It has potential far beyond its current use as a fringe technique only accessible to groups fortunate enough to be based in transplant centres. Routine use of this model holds great potential, and policy makers and funders should facilitate this new and exciting type of research.

## Conclusion

ESHP is a promising technology that extends beyond traditional heart transplantation, offering a platform for drug discovery and for testing novel therapies. It enables the evaluation of drug efficacy and safety in a controlled environment, thereby minimizing patient risk. However, challenges such as short perfusion times and logistical issues in obtaining hearts for research need to first be addressed. By overcoming these limitations, ESHP could revolutionize regenerative and genetic medicine and expand its applications to drug discovery, ultimately enhancing the treatment of heart failure and other cardiovascular diseases.

## Data Availability

The original contributions presented in the study are included in the article/Supplementary Material, further inquiries can be directed to the corresponding authors.
